# The Prevalence of Tail Alterations on German Dairy Farms

**DOI:** 10.3390/ani15182644

**Published:** 2025-09-09

**Authors:** Rieke Claussen, Roswitha Merle, Marina Volland, Stephanie Magnus, Kerstin-Elisabeth Müller

**Affiliations:** 1 Unit for Internal Medicine and Surgery, Farm Animal Clinic, Division for Ruminants and Camelids, School of Veterinary Medicine, Freie Universität Berlin, 14163 Berlin, Germany; marina.volland@fu-berlin.de (M.V.); stephanie.magnus@gmx.de (S.M.); kerstin-elisabeth.mueller@fu-berlin.de (K.-E.M.); 2 Institute of Veterinary Epidemiology and Biostatistics, School of Veterinary Medicine, Freie Universität Berlin, 14163 Berlin, Germany

**Keywords:** tail alteration, tail injury, dairy cow, animal welfare, deviated tail, shortened tail

## Abstract

This study aimed to determine herd prevalence of tail alterations (deviated and shortened tails) on farms in Germany. In total, 86,355 cows on 765 dairy farms in three regions (North, South, East) were assessed for presence or absence of deviated and shortened tails. Mean herd prevalence of deviated tails was 10.00% with a median of 7.89%. The mean herd prevalence of shortened tails was 1.07% with a median of 0%. Herd size, energy-corrected milk yield (ECM), and husbandry system were associated with herd prevalence rates of tail deviations and shortened tails. Herd prevalence increased with herd size (Spearman’s ρ = 0.586) and median ECM (ρ = 0.404). 28.10% farms had a herd prevalence <5% tail alterations, while 26.41% had a herd prevalence of >15%. Our findings plead for including monitoring of tail alterations in animal welfare assessments of dairy farms. The National Dairy FARM Program in New Zealand aims to achieve a herd prevalence of less than 5%. A herd prevalence of >15% tail alterations is regarded as the critical threshold requiring clarification and elimination of causes. It is imperative that all dairy farms strive to achieve a herd prevalence of less than 5% and welfare protocols are duly adjusted.

## Simple Summary

Injuries of the tail form an upcoming welfare issue in dairy cows. The cow’s tail is the extension of the spine and is well supplied with blood vessels and nerves. It fulfills important functions in animal communication and fly control. For this reason, the integrity of the tail has to be maintained. The present study aimed to determine the herd prevalence of tail alterations (deviations of the axis, shortened tails). In addition, herd size, median energy-corrected milk yield per farm (ECM), and the type of husbandry system were associated with herd prevalence rates of tail deviations and shortened tails. Tail tip alterations were not included in this observational study. Of 86,355 dairy cows scored on 765 farms, 11,602 had deviated tails, and 955 had shortened tails. Our observations reveal that on approximately 25% of farms, the herd prevalence of tail alterations was less than 5%, while on around 25% of the farms, the herd prevalence exceeded 15–52.4%. Our findings plead for including monitoring of tail alterations in animal welfare assessments of dairy farms. It is imperative that all dairy farms strive to achieve a herd prevalence of less than 5% and that welfare protocols are duly adjusted.

## 1. Introduction

Consumers of products originating from farm animals expect that, during their productive lives, these animals do not suffer from pain and discomfort [1,2]. The concept of the Five Freedoms forms the basis of today’s understanding of animal welfare. It refers to the freedom from hunger, pain, injury, disease, and the freedom to express species-specific behavioral patterns [3]. Although livestock husbandry has substantially improved in recent years, dairy cows still suffer from lesions caused by inappropriate housing conditions and improper animal handling. To fulfill the consumers’ demands, welfare-based quality assurance schemes with quality control ensured by on-farm independent audits were implemented [4,5]. Protocols to assist this process were developed, which include indicators providing information on housing conditions and space allowance, as well as animal-based indicators of animal welfare, such as lesions of the integument caused by collisions of the animals with the farm equipment or injuries originating from rough animal handling procedures [4,5].

The cow’s tail forms the extension of the spine. It consists of eighteen to twenty vertebrae and contains a large number of nerves and pain receptors [6]. The tail’s integrity is essential for the affective state of the dairy cow, which includes the ability to express the species-specific behavioral patterns as a sign of animal welfare [7,8]. The cow lifts its tail during urination and defecation [9,10,11]. Furthermore, the tail is a valuable instrument for communication with herd mates [9,10,11]. Moving its tail, the cow expresses her mood, indicates estrus, or signals the approaching parturition, another important function of the tail is to repel insects [9,10,11]. Various disorders of the bovine tail were reported [12,13,14]. Congenital malformations of the tail, such as the rat-tail syndrome, the crooked tail syndrome, and vertebral and spinal dysplasia [12,13]. These are either caused by gene defects or the exposure of the pregnant mother to toxins, malnutrition, or infectious agents [12,13].

The clinical appearance of traumatic tail injuries varies from superficial abrasions of the skin to deeper lesions associated with tissue swelling or deviations of the axis of the tail due to subluxation or luxation of vertebrae or tears of the caudal fascia, up to open or closed fractures [15,16]. Tail injuries impair fly protection and communication with herd mates [17]. They are painful and form a serious restriction on animal well-being [18,19].

Necrosis of the tail tip in dairy cows is a frequently occurring disorder [20]. Its etiology, however, has yet to be clarified [21]. Shortened tails are mostly the result of a surgical intervention (tail amputation) due to traumatic tail injury, tail tip necrosis, or tail docking.

Tail docking refers to the intentional prophylactic amputation of body parts in dairy cows, beef cattle, calves, and sheep [22]. Partial or total amputation of organs without a medical indication is forbidden in Germany [23]. In the United States of America (USA) and New Zealand (NZ), amputating the tail—the so-called tail docking—was routinely carried out to enhance the cow’s cleanliness and to assure the work safety of those employed in the milking process [24,25]. The idea underlying the procedure of tail docking is that the intervention reduces the risk of mastitis in dairy cows and protects the milking personnel from infections (leptospirosis) [25]. It was assumed that milking personnel getting hit by the tail switch, especially in the face, but also on the arms, is a potential risk factor for leptospirosis infections through urine contamination. [25]. In addition, tail docking was supposed to improve udder health. The latter assumption was refuted [26]. The prevention of leptospirosis can be achieved by wearing protective gloves and diagnostic serology [27]. There is no evidence-based justification for performing tail docking in dairy heifers [25].

Tail injuries and tail tip necrosis are frequent findings in beef cattle kept indoors on slatted floors [28,29]. Following Article 6 (3) of the German Animal Welfare Act, the veterinary authorities can permit the amputation of the lower end of the tail by use of rubber rings in male calves younger than three months. This intervention can only be performed if it is verified that improvement of housing conditions and management on the farm did not lead to a decrease in the prevalence of tail alterations [23]. The intervention results in an exposure of pain receptors and neuroma at the end of the tail, which prompts the animals to retract their tails towards the body, so the risk of stepping on the tail by other fattening bulls in the compartment should be reduced [19]. In fact, the intervention resulted in a decrease in the prevalence of tail alterations [19,30]. Furthermore, the painfulness of tail docking was examined [31]. In calves that were tail-docked by rubber rings, lying time was reduced, movements were increased, and rumination was reduced [24]. To determine chronic pain, the reactions to hot and cold stimuli and the observation of different surface temperatures in intact and amputated tails were compared to phantom limb pain in humans [32,33].

As prey animals, cattle express signs of weakness, discomfort, and pain as minimally as possible [9]. Consumers of food of animal origin and food producers demand evaluation of on-farm animal welfare by independent persons who apply standardized protocols that allow rating of the conditions under which farm animals are kept by applying validated resource-, management-, and animal-based indicators [1,34]. Only recently, tail injuries have been reported as a severe threat to animal welfare in dairy cows [35,36]. These are either a consequence of traumatization of the tail by the barn equipment or environmental factors (collision with the manure scraper or cubicle partitions), inappropriate handling procedures, or metabolic factors (subclinical acidosis) [15,37,38,39]. While the European Welfare Quality^®^ project developed standardized ways of assessing animal welfare and a standardized way of integrating this information to enable farms to be assigned to one of four categories (poor to good animal welfare) [40]. The information on “tail docking” solely applies to the surgical methods applied (nothing, rubber ring, surgery) and the use of medicines for pain relief [32,41].

This study aims at determining the prevalence of tail alterations (tail deviations, and shortened tails) on dairy farms in three different regions of Germany. Furthermore, the tail alterations were taken into account in the housing system, the herd size, and the milk yield, establishing benchmarks above which the herd prevalence of tail alterations, like deviated or shortened tails, needs clarification and abolishment of causes [16].

## 2. Materials and Methods

### 2.1. General Information

This study was conducted as part of a large cross-sectional study on health, biosecurity, and housing conditions on dairy farms in Germany [42]. The study was carried out by the veterinary faculties of three German universities: “University of Veterinary Medicine Hannover”, “Freie Universität Berlin”, and “Ludwig Maximilians University of Munich”, and was funded under grant number FKZ 2814HS006-008.

### 2.2. Dairy Farms

A total of 765 dairy farms distributed over three different regions across Germany were included in the study. Specifically, the North (Schleswig–Holstein, Lower Saxony) was represented by 253 farms, the East (Mecklenburg–Western Pomerania, Brandenburg, Thuringia, Saxony–Anhalt) by 252 farms, and the South (Bavaria) by 260 farms. The sample size per region was calculated assuming a prevalence of 40%, with a 95% confidence level, a power of 80%, and an accuracy of ±5%. Farms were pre-selected based on data obtained from the National Livestock Information Database (HIT) for the North and East, and from the Milchprüfring Bayern e.V. (Wolnzach, Germany) for the South. Eligible farms received a letter containing information on the aims and the design of the study and an invitation to participate. Farmers willing to participate in the study voluntarily contacted the respective study team and gave their written consent to participate in the study [42].

### 2.3. On-Farm Data Collection

Farm visits were carried out on a single occasion between December 2016 and August 2019. A total of 21 veterinarians from the three project partners collected data by interviews and clinical assessments of individual dairy cows. To assure the quality of data collection, study veterinarians received training in advance and in the course of the study. Intra-observer reliability of each study veterinarian and inter-observer reliability among the different study teams were evaluated for a number of indicators. All dairy cows per farm were examined up to a certain limit. The maximum was as follows: 130 cows in the South, 213 in the North, and 166 in the East. In the eastern region, 166 cows were examined per farm on farms with between 160 and 292 dairy cows, and 292 cows on farms with 293 dairy cows or more. Animals were selected at random and subsequently, if there was more than one distributed across all compartments of the farm. [42,43]. For identification and mapping of the data, the individual ear tag number of each animal (last five digits) was recorded [44].

The farming and housing systems were recorded (e.g., flooring type, cubicle dimensions, access to pasture). The assessment of the cows included cleanliness, technopathies, height, body condition score, locomotion score, deviation or shortening of the tail, and rip swellings. In addition, farmers were interviewed during the farm visit to collect further information on farm characteristics (full-time or part-time farming, organic or conventional farming). Data on milk yield, parity, age, breed, and days in milk were retrieved for individual cows from the National Animal Information Database (HIT) and the National Milk Recording System (DHI). Farm records of milk yield were available for individual cows up to 12 months prior to the farm visit from those 621 farms that gave the respective consent. Milk yield and milk composition were assessed once a month. The median of energy-corrected daily milk yield (ECM) data over the last 12 months was used in this study. ECM was calculated according to the description provided by Sjaunja et al. in 1990 [45].

### 2.4. Clinical Inspection of the Tail and Categorization of Alterations

Dairy cows were assessed for the presence of visible alterations at the tail. Deviated tails refer to tails that are not straight but rather those that are bent or angled in an unnatural direction. Shortened tails were supposed to be related to tail amputations performed in the past due to traumatic lesions or ascending infections of the tail, due to tail tip necrosis. The criteria applied were categorized as follows: 1 = no deviation/swelling of the tail, no shortened tail, 2 = deviation or swelling of the tail, 3 = shortened tail. Tail tip alterations were not considered in the present study, but were reported elsewhere [21].

### 2.5. Data Handling and Statistical Analysis

All data were collected using standardized questionnaires and data entry forms. After the site visit, data were manually entered into a central Structured Query Language (SQL) database. Descriptive statistics were performed using IBM^®^ SPSS^®^ Statistics “ Version 29.0.0.0 (IBM Deutschland GmbH, 71139 Ehningen, Germany).

Since there were significant disparities in husbandry conditions across the different regions, a separate analysis for each region was needed.

Herd prevalence of deviated and shortened tails were calculated by dividing the respective number of affected animals by the number of investigated animals. Descriptives, including means and medians, were calculated over farm-level prevalence values. The means were calculated as unweighted means.

The herd prevalence of tail injuries was analyzed in association with the ECM on the different farms. For each farm, the median of the ECM of individual dairy cows from test results over the last 12 months was collected and averaged per farm. The categories were defined as ≤24 L ECM, 25 to 30 L ECM, and >30 L ECM. Herd size was categorized into <60, 60–119, ≥120 dairy cows to obtain nearly similar group sizes in small and large herds.

The predominant housing system was defined as that housing system in which at least 80% of the cows were kept on the day of the farm visit. The farms were classified into one of the five categories: loose housing with cubicles, straw–based free stalls (parts of walking and all lying areas are scattered with straw, with no cubicles dictating a specific lying position), tie–stalls, and pasture–based housing systems.

Spearman’s correlation coefficients (ρ) were calculated to examine the correlation between herd prevalence of deviated tails, herd size, and deviated tails and ECM (both on a continuous scale), and statistically classified [46].

## 3. Results

In total, 86,355 cows on 765 dairy farms were assessed and categorized for the presence or absence of deviated and shortened tails at the farm level. 11,602 (13.44%) dairy cows had deviated tails, and 955 (1.11%) had shortened tails. The mean herd prevalence of deviated tails was 10.00%, with a median of 7.89% per farm. Across all regions, the observed herd prevalence ranged from 0.00% to a maximum of 52.35% (Table 1).

The mean herd prevalence of shortened tails was 1.07%, with a median of 0% and a range from 0.00% to 20.42% at the farm level (Table 2).

**Figure 1:**
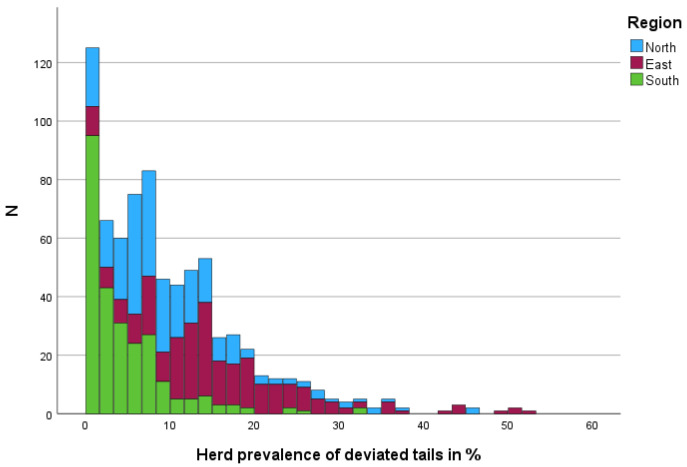
Distribution of farms (N) by herd prevalence of deviated tails in %, stratified by region.

In total, there was a mean herd prevalence of 11.07% for tail alterations observed on dairy farms over the three different regions in Germany. On 98 (12.8%) of farms, no cow with visible tail alteration (deviated and shortened tail) was observed (Figure 1). Ten of these farms were located in the East, twelve in the North, and 76 in the South.

On 112 (14.64%) of the farms no deviation of the tails was observed. Approximately 17 farms were in the North, 10 farms in the East, and 86 farms with no deviated tails in the observed cows were in the South.

**Figure 2:**
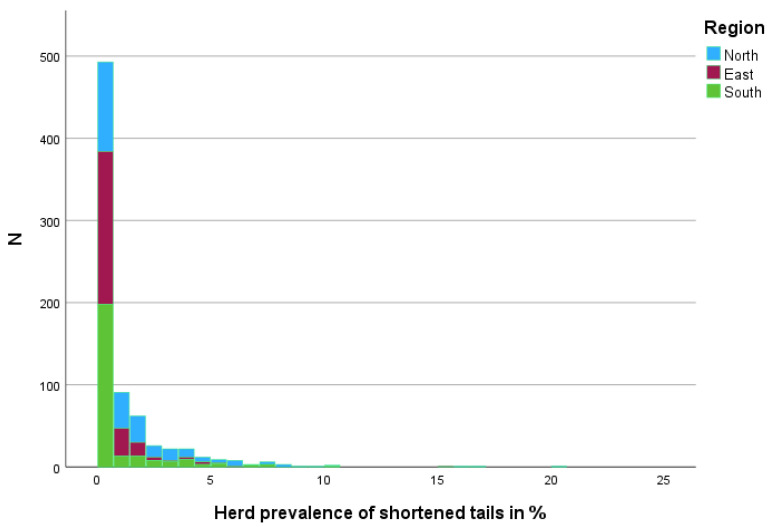
Distribution of farms (N) by herd prevalence of shortened tails in % stratified by region.

On 416 (54.38%) farms, no shortened tail was recorded (Figure 2) (98—North, 120—South, 198—South).

Herd prevalence of tail deviation of less than 1.00% was recorded for 14.77% (113 farms) of the dairy farms (18—North, 10—East, 86—South).

A total of 215 (28.10%) farms had less than 5% cows with tail alterations (deviated and shortened tails) (41—North, 22—East, 152—South). A less than 15% herd prevalence was observed in 563 farms (73.59%; 190—North, 130—East, 190—South). With a herd prevalence of less than 15% among the observed cows, 190 of the farms were observed in the North, 130 farms in the East, and 190 farms in the South.

### 3.1. Prevalence of Tail Alterations By Herd Size

The median herd prevalence of deviated tails was lowest on farms with a herd size <59 dairy cows (3.48%) and highest in herds with >120 dairy cows (13.97%) (Table 1).

On a continuous scale, deviated tails and herd size were correlated with a Spearman correlation coefficient of ρ = 0.586.

For shortened tails, the highest herd prevalence of 0.98% (median) was observed on farms with 60 to 119 cows and 0.57% in herds of more than 120 dairy cows (Table 2).

### 3.2. Prevalence of Tail Alterations on Farms ECM

The prevalence of tail injuries was analyzed in association with ECM on the different farms.

The median herd prevalence of deviated tails was lowest on farms with up to 24 L ECM (4.4%) and highest on farms with a milk yield > 30 L ECM (12.5%) (Table 1). Also, on a continuous scale deviated tails and median ECM correlated positively (ρ = 0.404).

For shortened tails, median herd prevalence was lowest for ≤24 L ECM milk yield (0%), and the highest for >30 L ECM milk yield (0.56%) (Table 2).

### 3.3. Prevalence of Tail Alterations Depending on the Housing Systems

Farms providing loose housing systems for dairy cows had the highest herd prevalence of deviated tails. In all regions, the median herd prevalence of deviated tails in free stalls with cubicles (8.54%) and straw–based systems (7.41%) was very similar. Concerning the regions, the herd prevalence of deviated tails ranged from a median of 3.45% (South) to a median of 8.2% (North) and a median of 14.56% (East) for free stalls with cubicles (Table 1).

Farms with pasture–based systems and those with tie–stalls had the lowest overall prevalence of deviated tails.

In tie–stalls, a greater regional heterogeneity was observed with the lowest values in the South (0.00%) and the highest in the North (8.00%).

For farms classified into the mixed category, the median prevalence of deviated tails for all regions was 9.93%. (Table 1).

Concerning shortened tails, the highest median herd prevalence was observed on farms with loose housing with cubicles (0.33%) and mixed housing systems (0.33%) (Table 2). The highest regional herd prevalence of shortened tails was observed in the South for straw–based free stalls (median of 5%). Only two farms with straw–based free stalls were included in this region. The lowest prevalence of shortened tails in straw–based free stalls was observed in the East (median of 0%). For free stalls with cubicles, the highest median herd prevalence of 1.08% in the North, and the lowest at 0.33% in the East.

On farms with pasture–based housing systems and tie–stalls, the lowest overall mean herd prevalence of shortened tails was determined with 0.72% in pasture–based systems and 0.54% in tie–stalls. The median of shortened tails in tie–stalls and pasture–based systems for each region and in total is 0% (Table 2).

**Table 1:** Description of herd prevalence of deviated tails in % observed on dairy farms in three regions of Germany (North, East, and South) on a single farm visit.

			N	Mean	Median	SD	Min	0.25	0.75	Max
Region		North	253	9.97	8.03	7.83	0.00	5.29	13.02	46.55
		East	252	15.67	14.06	9.65	0.00	9.9	19.87	52.35
		South	260	4.54	3.01	5.53	0.00	0.00	6.94	33.33
		Total	765	10.00	7.89	9.05	0.00	3.35	14.1	52.35
Herd size ^1^		≤59	292	5.43	3.48	6.54	0.00	0.00	7.89	37.5
		60–119	203	9.5	7.46	8.91	0.00	4.08	12	51.67
		≥120	270	15.33	13.97	8.69	0.71	9.17	19.26	52.35
ECM in L ^2^		≤24	166	6.07	4.4	6.71	0.00	0.00	8.6	44.44
		25–30	320	10.31	8.2	8.41	0.00	3.88	14.56	46.55
		>30	135	14.69	12.5	9.69	0.00	7.95	18.92	51.67
Predominant Housing system ^3^	North	Tie–stall	9	8.37	8	5.37	2.38	2.79	13.25	17.39
		Loose housing with cubicles	211	10.3	8.2	7.9	0.00	5.61	13.21	46.55
		Straw–based	6	11.29	8.61	11.49	0.00	4.41	16.82	33.33
		Pasture–based	9	4.8	4.35	5.47	0.00	0.00	8.33	15.56
		Mixed	18	8.95	6.02	7.38	0.00	3.54	11.85	27.17
	East	Tie–stall	3	15.88	7.5	18.88	2.63	2.63	–	37.5
		Loose housing with cubicles	198	16.85	14.56	9.51	0.00	11.05	20.53	52.35
		Straw–based	11	10.17	6.67	9.77	0.00	0.00	21.18	25.54
		Pasture–based	6	3.13	0.00	5.23	0.00	0.00	7.81	12.5
		Mixed	34	12.75	12.73	7.61	0.00	7.29	16.98	30.57
	South	Tie–stall	77	3.34	0.00	5.41	0.00	0.00	5.13	33.33
		Loose housing with cubicles	175	4.94	3.45	5.38	0.00	1.33	7.14	32.86
		Straw–based	2	7.73	7.73	4.78	4.35	4.35	–	11.11
		Pasture–based	0	–	–	–	–	–	–	–
		Mixed	6	7.3	3.81	9.41	0.00	0.00	14.99	24.24
	Total	Tie–stall	89	4.27	2.08	6.56	0.00	0.00	7.14	37.5
		Loose housing with cubicles	584	10.92	8.54	9.19	0.00	4.44	14.76	52.35
		Straw–based	19	10.27	7.41	9.59	0.00	2.70	17.14	33.33
		Pasture–based	15	4.13	0.00	5.25	0.00	0.00	7.14	15.56
		Mixed	58	11.01	9.93	7.88	0.00	5.11	14.85	30.57

N: Number of farms; SD: standard deviation; Min: minimum; Max: maximum; ^1^ categorized by number of dairy cows; ^2^ median of the energy-corrected daily milk yield (ECM) from test results of the last 12 months; ^3^ >80% of the cows kept in the respective housing system on the day of the farm visit.

**Table 2:** Description of herd prevalence of shortened tails in % observed on dairy farms in different regions of Germany (North, East, and South) on a single farm visit.

			N	Mean	Median	SD	Min	0.25	0.75	Max
Region		North	253	1.80	0.98	2.72	0.00	0.00	2.28	20.42
		East	252	0.66	0.32	1.35	0.00	0.00	0.81	15.48
		South	260	0.75	0.00	1.65	0.00	0.00	0.00	10.00
		Total	765	1.07	0.00	2.06	0.00	0.00	1.34	20.42
Herd size ^1^		≤59	292	0.80	0.00	1.83	0.00	0.00	0.00	10.00
		60–119	203	1.48	0.98	2.34	0.00	0.00	1.80	16.95
		≥120	270	1.04	0.57	2.01	0.00	0.00	1.09	20.42
ECM in L ^2^		≤24	166	0.84	0.00	1.75	0.00	0.00	0.93	8.45
		25–30	320	1.14	0.33	1.91	0.00	0.00	1.59	15.48
		>30	135	1.03	0.56	1.53	0.00	0.00	1.21	7.69
Predominant Housing system ^3^	North	Tie–stall	9	0.60	0.00	1.20	0.00	0.00	1.28	2.86
		Loose housing with cubicles	211	1.89	1.08	2.83	0.00	0.00	2.25	20.42
		Straw–based	6	0.60	0.00	1.46	0.00	0.00	0.89	3.57
		Pasture–based	9	1.20	0.00	2.42	0.00	0.00	2.27	6.25
		Mixed	18	2.05	1.35	2.34	0.00	0.00	3.74	8.00
	East	Tie–stall	3	0.81	0.00	1.41	0.00	0.00	–	2.44
		Loose housing with cubicles	198	0.68	0.33	1.42	0.00	0.00	0.83	15.48
		Straw–based	11	0.32	0.00	0.60	0.00	0.00	0.57	1.75
		Pasture–based	6	0.00	0.00	0.00	0.00	0.00	0.00	0.00
		Mixed	34	0.78	0.33	1.18	0.00	0.00	1.10	4.76
	South	Tie–stall	77	0.52	0.00	1.60	0.00	0.00	0.00	7.69
		Loose housing with cubicles	175	0.77	0.00	1.46	0.00	0.00	1.25	7.14
		Straw–based	2	5.00	5.00	7.07	0.00	0.00	–	10.00
		Pasture–based	0	–	–	–	–	–	–	–
		Mixed	6	1.57	0.00	2.82	0.00	0.00	3.57	6.98
	Total	Tie–stall	89	0.54	0.00	1.55	0.00	0.00	0.00	7.69
		Loose housing with cubicles	584	1.14	0.33	2.13	0.00	0.00	1.47	20.42
		Straw–based	19	0.90	0.00	2.38	0.00	0.00	0.57	10.00
		Pasture–based	15	0.72	0.00	1.93	0.00	0.00	0.00	6.25
		Mixed	58	1.26	0.33	1.87	0.00	0.00	2.04	8.00

N: Number of farms; SD: standard deviation; Min: minimum; Max: maximum; ^1^ categorized by number of dairy cows; ^2^ median of the energy-corrected daily milk yield (ECM) from test results of the last 12 months; ^3^ >80% of the cows kept in the respective housing system on the day of the farm visit.

## 4. Discussion

Observations on 86,355 dairy cows on 765 farms revealed a mean herd prevalence of visible tail alterations of 11.07%. Although the husbandry systems vary widely between New Zealand (NZ), Ireland, and Germany, with pasture–based hybrid systems, spring calving in South Ireland [36], and pasture–based systems in NZ [16,37], data from NZ and Ireland demonstrate prevalence rates for tail injuries of 9.2% to 11.5% [16,36,37], which is in accordance with the findings of our study. Lower prevalence was reported from the US, with 3.1% for deviated tails and 6.7% for shortened tails in tie–stalls [47].

The national animal welfare program in NZ sets a maximum limit of 5% for tail damage [48]. In Germany, only 28.1% of dairy farms were below this benchmark. In NZ, a prevalence of >15% is regarded as the critical threshold above which dairy farms urgently need to take appropriate measures [16]. On 26.41% of the dairy farms included in the present study, the herd prevalence of tail alterations, excluding tail tip necrosis, exceeded 15%. While the attention to tail alterations was directed at fattening bulls in the past [19,28,29], the latter percentage clearly pleads for the need of monitoring tail alterations in dairy cows and performing causal research on German dairy farms. In 98 of the 765 farms, no tail alterations were found. The distribution of results was found to be heterogeneous, underlining the necessity of evaluating potential risk factors underlying these substantial variations. Such an evaluation should particularly take into account structural differences and the predominant husbandry conditions across farms.

### 4.1. Prevalence of Deviated Tails as a Consequence of Herd Size, Housing Conditions, and Performance Level of the Herd

Only 14.64% of the study farms had no cows with a deviated tail. In Europe, only the UK Royal Society for the Prevention of Cruelty to Animals included in their welfare standards for dairy cattle that the absence of deviated tails is a positive sign of dairy cow welfare on farms with good management [49]. Tail alterations belong to the category of technopathies [49]. The term technopathy refers to health issues or injuries cows experience due to interactions with farm equipment, facilities, and farm workers in dairy farming, such as hairless areas and decubitus at the legs, as well as lameness, that should be observed and prevented [49]. It has been proposed that a prevalence of less than 1.00% for deviated tails should be considered a possible benchmark in the context of dairy cow husbandry [50]. It is recommended that greater focus should be placed on deviated tails and that these should be integrated into welfare protocols, because the mean herd prevalence of 10.00% for deviated tails, with an on-farm range of 0–52.35%, is consistent with the findings of previous studies [16,37]. In our study, a prevalence of less than 1.00% was recorded in 14.77% (113 farms) of the dairy farms included in the study. It is evident that this is below the specified 70% threshold for pasture–based husbandry [50].

The herd prevalence of deviated tails showed a moderate correlation with milk yield and herd size [46]. These findings suggest that management and/or husbandry factors may increase the risk of tail injuries in dairy cows. Twisting of the tail by farm workers in order to force the animal to move faster to the milking parlor is assumed to cause luxation of vertebrae or rupture of the fascia that keeps the vertebrae in place. The force required to actually break a tail is 9.8–20 newton-meter (Nm) [15]. The torque of 9.8 Nm exerted on the human shoulder joint when lifting a weight with your outstretched arm, the required weight can be determined. For a distance of 60 cm between the shoulder and hand, the required weight is 1.66 kilograms (kg). In order to generate a torque of 20 Nm on the shoulder joint, it is necessary to apply a weight of 3.4 kg. This force is unlikely to be exerted unintentionally when a cow’s tail is lifted carefully and controlled in this manner [15]. Unfortunately, Spearman’s correlation could only be conducted for these two variables. This is because continuous data were required but not available for the predominant housing system. Likewise, the number of shortened tails was significantly lower. As a result, correlation analysis was precluded in this case as well.

In tie–stalls, the height of the tie-rail was shown to be positively correlated with the occurrence of deviated tails and negatively correlated with the cleanliness of the hind limbs [38]. There is currently no study available that considers the association of husbandry conditions in loose housing and management factors with tail injuries. The herd prevalence rates found in this study indicate that the causes of deviated tails should be further explored. Additionally, there is no data for the estimation of pain due to injured or inflamed tails of cattle. Since deviated tails form an animal welfare issue [48,49], these should be included in welfare assessment protocols for dairy farms.

### 4.2. Prevalence of Shortened Tails Depending on Management Factors

Shortened tails are attributed to severe trauma and necrosis following ischemia of the tip of the tail [14]. The present study of herd prevalence for shortened tails shows a mean value of 1.07 and a median of 0. However, some farms had a high herd prevalence of up to 20.42%. Significant regional variations were observed for both shortened and deviated tails. Amputated tails are associated with pain [18,30]. Thus, despite the low herd prevalence, it is essential to carry out a thorough analysis to identify the underlying causes.

Studies on tail tip necrosis were carried out on beef cattle farms, where overstocking, leukocytosis, and hyperproteinemia were associated with tail tip necrosis [29]. The beef cattle in the study of Kordowitzki, 2015 [29], are mostly fed a high-energy diet with high amounts of starch, which leads to subacute rumen acidosis (SARA). The acidic pH value, as well as the space allowance, were identified as significant risk factors for tip-tail necrosis.

To our knowledge, there are no studies investigating the risk factors for shortened tails in dairy cows. In some studies, various types of tail lesions were found—such as lesions on the very tip of the tail, wart-like masses, and annular constrictions—which are also called band-shaped tail (BST) lesions [20,21]. The BST lesion causes ischemia, followed by necrosis and subsequent loss of the distal part of the tail. Aside from the amputation of the tail, this is another possible cause for a shortened tail in our study [14].

### 4.3. Limitations of the Study

The population of the present study represents the heterogeneous types of dairy cow farming in different regions of Germany, which covers different farm types and some risk factors might apply only to a part of the farms. Although farms were randomly selected and participated on basis of voluntariness. Farmers with more pronounced problems may not have answered the invitation to the study, which might have led to a bias with respect to farmers who are engaged in the improvement of animal welfare conditions.

Based on the available data, it is not possible to attribute shortened tail to necrosis or trauma that indicated amputation.

## 5. Conclusions

The mean herd prevalence of tail injuries, which has been recorded at 11.07%, is comparable with the findings of other authors. At present, there is no national animal welfare protocol for the assessment of dairy farms in Germany that includes the evaluation of tail alterations. Due to the pain sensation associated with tail injuries in the acute phase and the potential inability to use the tail as it should in chronic stages, tail injuries form a welfare issue on dairy farms. There seem to be different causes underlying deviated and shortened tails.

The present analysis indicates a moderate correlation between milk yield and herd size on the one hand, and tail injuries on the other. However, the data suggest that different risk factors need to be associated with deviated and shortened tails. Given the fact that the prevalence of tail alterations exceeds 15% on 26.41% of farms, it is imperative that, besides adding assessment of tails to animal welfare protocols for dairy farms, a detailed analysis of the risk factors for tail injuries has to be conducted as a matter of urgency. This will provide farmers with the means to address this issue.

## Data Availability

The datasets presented in this article are not readily available because the data were acquired through cooperation between different universities. These universities signed a cooperation contract and agreed that any data transfer to interested persons is not allowed without an additional formal contract. Data are available for qualified researchers who sign a contract with the project consortium. This contract will include guarantees of the obligation to maintain data confidentiality in accordance with the provisions of German data protection law. Currently, there exists neither a data access committee nor another body that could be contacted for the data; a committee will be founded for this purpose. This future committee will consist of the authors as well as members of the related universities. Requests to access the datasets should be directed to the following: A. Campe, Institute of Biometry, Epidemiology, and Information Processing at the University of Veterinary Medicine, Hannover, Bünteweg 2, 30559 Hannover, Germany, Email: anely.campe@tiho-hannover.de; M. Hoedemaker, Clinic for Cattle at the University of Veterinary Medicine, Hannover, Bischofsholer Damm 15, 30173 Hannover, Germany, Email: martina.hoedemaker@tiho-hannover.de; R. Merle, Institute of Veterinary Epidemiology and Biostatics at the School of Veterinary Medicine at the Free University Berlin, Königsweg 67, 14163 Berlin, Germany, Email: roswitha.merle@fu-berlin.de.
